# Blood test result communication in primary care: mixed-methods systematic review protocol

**DOI:** 10.3399/BJGPO.2023.0105

**Published:** 2023-10-04

**Authors:** Helen Nankervis, Alyson Huntley, Penny Whiting, William Hamilton, Hardeep Singh, Sarah Dawson, Jane Sprackman, Anna Ferguson Montague, Jessica Watson

**Affiliations:** 1 University of Bristol, Bristol, UK; 2 University of Exeter, Exeter, UK; 3 Baylor College of Medicine, Houston, USA

**Keywords:** Blood tests, hematological tests, patient-centred care, patient satisfaction, communication

## Abstract

**Background:**

After testing, ensuring test results are communicated and actioned is important for patient safety, with failure or delay in diagnosis the most common cause of malpractice claims in primary care worldwide. Identifying interventions to improve test communication from the decision to test through to sharing of results has important implications for patient safety, GP workload, and patient engagement.

**Aim:**

To assess the factors around communication of blood test results between primary care providers (for example GPs, nurses, reception staff) and their patients and carers.

**Design & setting:**

A mixed methods systematic review including primary studies involving communication of blood test results in primary care.

**Method:**

The review will use a segregated convergent synthesis method. Qualitative information will be synthesised using a meta-aggregative approach, and quantitative data will be meta-analysed or synthesised if pooling of studies is appropriate and data are available. If not, data will be presented in tabular and descriptive summary form.

**Conclusion:**

This review has the potential to provide conclusions about blood test result communication interventions and factors important to stakeholders, including barriers and facilitators to improved communication.

## How this fits in

After testing, ensuring test results are communicated and actioned is important for patient safety, with failure or delay in diagnosis the most common cause of malpractice claims in primary care worldwide. Identifying interventions to improve test communication from the decision to test through to sharing of results has important implications for patient safety, GP workload, and patient engagement.

## Introduction

Blood tests are important for diagnosis and monitoring, but tests in themselves do not make people better, unless actions based on the test result lead to a change in patient management or reassurance. Both are dependent on test communication, which requires clear systems and processes for information sharing before and after blood testing.

A systematic review of US studies quantifying failures in test result follow-up has shown that between 6.8% and 62% of laboratory tests are not followed up; no relevant UK research was identified.^1^ Surveys and focus group studies have shown that UK general practices generally rely on patients contacting the practice to obtain their test result, with a lack of fail-safe mechanisms.^2–4^

Safe and efficient systems of test result communication are important in the current context of rising primary care workload,^5^ with the average GP estimated to spend 1.5–2 hours per day reviewing and actioning test results.^6^ Recent advances in IT systems, such as text messaging or online patient access to results, offer potential to improve test result communication, and NHS case studies have suggested these may reduce primary care workload,^7^ but evidence to back up these claims is lacking. In England, the NHS has started rolling out online access to blood test results to patient by default, via the NHS App and other online services. This is part of a wider move towards transparency and openness in health care; however, mixed methods studies have found that patients face challenges and confusion when viewing their test results,^8^ which are currently not presented in a patient-centred way. The James Lind alliance identified as a priority the need to provide information in patient medical records in a way that improves safety and quality of care.^9^

The study protocol has been registered on PROSPERO (Registration Number CRD42023427433) and is reported according to the PRISMA-P guidelines.^10^

### Aims

To assess the factors around communication of blood test results between primary care providers (for example GPs, nurses, reception staff) and their patients and carers.

### Objectives

This study aims to answer the following research questions:

1. What interventions can be used to improve communication of blood test results to patients and carers in primary care?
2. What are patients’ and carers’ needs and preferences for blood test result communication?
3. What are the needs and preferences of primary care staff and providers when communicating blood test results?
4. What are the barriers and facilitators to successful communication of blood test results?

### Eligibility criteria

Primary studies of any design, except case studies, that provide information on the communication of blood test results by primary care staff (for example doctors, nurses, physiotherapists, receptionists) and providers (for example primary care practices, primary care networks, medical health insurance providers) to adult patients and carers will be eligible for inclusion. As ‘primary care’ varies across the world and has no single agreed definition,^11^ the authors will include all studies except those on emergency, urgent, or acute care or where the participants were inpatients. This will allow the authors to review all available evidence relevant to primary care provision. If the number of included studies is unfeasible using these criteria, then the authors will restrict this to studies where a primary care population was described and the terms accepted in the full review report will be reported.

**Figure 1.**
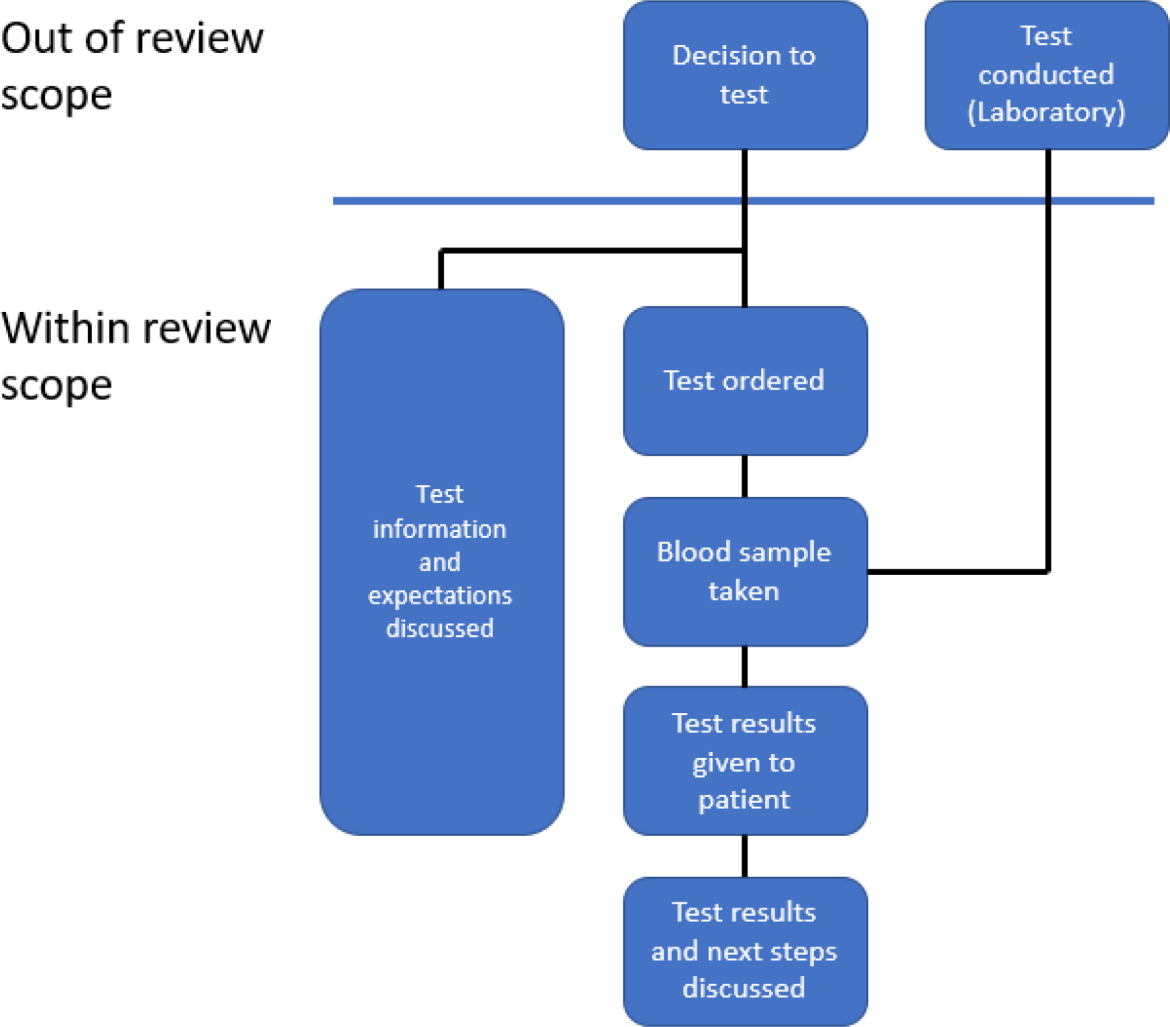
Study inclusion and exclusion flowchart

The authors define ‘communication of blood test results’ as any communication from the time of agreeing to order a test onwards, including what to expect from the results; when to expect the results; conveying the test results; how to interpret and understand the results; and conveying and understanding the next steps. This includes the systems within primary care that are aimed at ensuring communication of blood test results to patients and carers takes place. The authors will include studies where artificially generated data or hypothetical scenarios were used. Figure 1 shows the review's inclusion and exclusion flowchart.

Studies that focus exclusively on point-of care tests (this includes self-administered tests, such as blood glucose testing), genetic tests or communication of test results from laboratories to primary care, and studies of blood tests exclusively in children will also be excluded.

## Method

### Search strategy

The authors will use an iterative and flexible approach to the searches, to find both quantitative and qualitative research.

The authors will search the following databases: Medline (Ovid), Embase (Ovid), PsycINFO (Ovid), CINAHL (ESCOHost), and the Cochrane Library for primary studies. They will also hand search the reference lists of eligible full texts. In addition, the authors will search the grey literature, including NHS websites and will contact experts in the field, as they anticipate that some relevant literature will be published as quality improvement reports rather than formal research. The search will be restricted to 2013 onwards to keep the review relevant, as communication knowledge, interventions, and technologies develop rapidly.

Many of the key search terms are generic, frequently occurring words in the biomedical and healthcare literature, therefore a set of known studies (gathered from experts in the field and informal scoping searches) will be used to help seed the full search strategy. Elements of cluster searching and key pearl citations to find other ‘kinship’ studies will be used.^12^ The main complementary search techniques will be citation searching (forwards and backwards), following lead authors and related projects. These studies will be used to help create a list of the most relevant key words and database subject headings. The initial MEDLINE search created using the methods outlined above (see Appendix 1) will be adapted, optimising it for use in the other search databases, taking into account their size, functionality, and subject coverage. Searches will be amended and rerun if the authors believe that it will be beneficial to do so, after identifying any new search terms throughout the process of screening and study selection.

### Study selection

EndNote (version 20) will be used to save and de-duplicate search results, and the total number of results before and after de-duplication for each database searched will be recorded. Two reviewers will independently screen titles and abstracts identified by the searches using Rayyan.^13^ Full copies of all reports considered potentially relevant will be obtained, and two reviewers will independently assess these full texts for inclusion. Any disagreements will be resolved by consensus or discussion with a third reviewer. Any studies excluded at this stage will be recorded with the reason for exclusion.

### Data extraction

Data will be extracted using forms, which will be jointly developed by the team, piloted on a small sample of studies, and adapted iteratively as necessary. One reviewer will extract data, and these will be checked by a second reviewer. Study authors will be contacted for clarification, where data needed for the review are not available in the report.

Interventions will be subdivided according to a framework developed by Singh *et al* ^14^ into interventions targeting: 1) interpersonal communication, defined as the verbal exchange of information between primary healthcare staff and patients; and 2) informational communication, defined as written instructions or laboratory values shared by text message, email, online portal, or printed text or leaflets.

The authors anticipate that the data extraction form will include (but not be limited to) the following study characteristics:

- geographical location of study population,
- healthcare system(s) the study is conducted in,
- study aims,
- sample size,
- study design,
- details of test(s) studied,
- mode of delivery of test results,
- type of intervention studied,
- barriers and facilitators to test communication,
- outcomes reported,
- results of study, where applicable.

### Quality assessment of included studies

The authors anticipate that the included studies will be of different designs. Risk of bias will be assessed for quantitative studies that aim to assess the benefits and harms of an intervention and the quantitative component of mixed-methods studies, using the RoB 2 tool^15^ for randomised controlled trials and ROBINS-I tool^16^ for non-randomised studies of interventions. The ROBINS-E tool^17^ will be used for non-randomised studies of exposure. These tools were chosen for consistency of toolset when assessing risk of bias.

Qualitative studies and the qualitative component of mixed-methods studies will be assessed using the JBI tool for qualitative studies.^18^

Where the study design does not fit into the designs detailed above, these will be discussed as a team and a suitable quality assessment tool chosen on a case by case basis, if available. Where a suitable tool does not exist, the authors will highlight the key strengths and weaknesses. All eligible studies will be included, regardless of the results of the quality assessments, and the information on quality will be used when drawing conclusions about the evidence.

### Data synthesis and integration

This synthesis will be conducted following the guidance of the JBI for mixed-methods systematic reviews using a segregated convergent approach.^19^ In this approach, quantitative and qualitative evidence are first evaluated separately using a segregated approach to synthesis, followed by (if appropriate, based on the data) a mixed-methods convergent synthesis to combine the quantitative and qualitative evidence. This approach, as opposed to a convergent integrated approach, has been chosen as the authors do not know in advance whether both qualitative and quantitative evidence can be used to answer each of their review questions. If it is not appropriate to conduct the mixed method convergent synthesis of the evidence, then the authors will summarise the results of the two segregated syntheses in this review or they may choose to report the two syntheses as separate reviews.

The authors will report and explore care setting, healthcare system, and mode of delivery of test results and outcomes, where this information is available. They will report any gaps in the evidence on blood test communication where they identify them from synthesising and integrating the evidence.

### Synthesis of qualitative evidence

The authors will use the meta-aggregative approach to qualitative synthesis, following the JBI guidance.^20,21^ This pragmatic method involves extracting study findings, often as a direct quote, then creating categories of findings, and, if possible, pooling the categories of findings into synthesised findings. Synthesised findings aim to convey the overall meaning of the categorised findings via statements. This approach is a good fit for systematic reviewing and will enable the authors to produce generalisable recommendation statements aimed at guiding practitioners and policy makers without seeking to re-interpret the primary studies’ findings.

The meta-aggregative approach uses only the primary study author’s findings in the aggregation of information from the studies. The authors will also look at the primary study evidence to identify any themes that relate to the research questions that have not been dealt with in the author’s findings. Using a thematic approach^22^ in addition, if necessary, ensures that the authors will be able to use all relevant information available.

### Synthesis of quantitative evidence

If the authors find two or more studies on the same intervention with the same outcomes, they will conduct meta-analyses to estimate summary measures of effect. They will calculate the mean difference between groups with 95% confidence intervals for continuous outcome data and relative risk with 95% confidence intervals for dichotomous outcomes, where possible. The intention for this review is to generalise the results of any meta-analyses beyond the studies included, therefore, a random effects model will be the default choice of statistical model. The authors will consider applying a fixed effects model if only five or fewer studies can be included in a meta-analysis and/or statistical and study characteristics (for example, population, setting, proposed mechanism of action of the interventions) heterogeneity are low, suggesting a common underlying effect.^23^ The authors will assess the heterogeneity of studies using the X^2^ and I^2^ statistics. Prior to conducting a meta-analysis, they will create a detailed analysis plan.

The authors anticipate that the included studies are highly unlikely to test the same interventions and use the same outcomes, making meta-analyses inappropriate. Where meta-analysis is inappropriate or not possible, they will synthesise the findings using the following narrative synthesis methods. Included studies will be grouped by intervention type within summary tables. Where they find studies reporting effect estimates, they will present these using median, quartiles, and range, if possible, in the tabular summary and as a bubble (small numbers) or box and whisker graphs (larger numbers). Where studies do not report effect estimates, they will report the results presented in the tables and summarise these descriptively.^24^ They will report these narrative syntheses using the SWiM guidance,^25^ which promotes transparent reporting of narrative synthesis methods using nine key reporting items.

### Mixed-methods (convergent) synthesis

The authors will attempt to integrate the separate qualitative and quantitative evidence syntheses using juxtaposition and organisation to answer the review’s research questions in a configured analysis. If configuration is not appropriate, then the findings of the qualitative and quantitative evidence will be reported separately, using tabular and descriptive summaries to address each research objective of this review. The authors will 'qualitise' the quantitative evidence, which involves translating it into textual descriptions that can be integrated with qualitative data. This is less prone to error than 'quantising' qualitative evidence by ascribing numbers to the descriptions and themes found.^19^

### Patient and public involvement

Patient and public involvement participants are co-authors on this protocol and and will be involved as co-authors for the full review. A patient and public involvement group has provided input into the design of the protocol and will be consulted for the review on: the emerging results; the dissemination strategy; the review findings; and future research plans, in order to address gaps in the current literature.

## Discussion

### Summary

Communication of blood test results in primary care has not previously been reviewed as a whole topic. Previous evidence syntheses have concentrated on specific types of communication interventions,^26^ on point of care testing,^27,28^ or for a particular condition or testing as a whole.^29,30^

### Strengths and limitations

By involving patient and participant representatives in the design of this review, the authors are ensuring that the questions being asked are important to primary care users as well as primary care providers and researchers. The evidence base is heterogeneous, so it is unlikely that this review will be able to synthesise large numbers of studies to help to answer the research questions posed. The area of blood test result communication has a broad scope and collection of terms used to describe it, making the search for relevant studies particularly challenging.

### Implications for research and practice

This systematic review will provide an overview of the existing research on communication of blood test results and where there are gaps in the evidence. The review will map out the current evidence, to help understand which types of blood tests have been studied, what methods and interventions have been used, and the barriers to implementation in primary care.

The evidence from this review can be used when developing future studies aimed at improving the test result communication process. Outcomes from this research will be important for patients, healthcare professionals, and healthcare systems, potentially improving patient outcomes and reducing primary care workload.

## Dissemination

We will aim to publish the results of this review in an open access journal.
